# Holistic Strategies to Control *Salmonella* Infantis: An Emerging Challenge in the European Broiler Sector

**DOI:** 10.3390/microorganisms11071765

**Published:** 2023-07-06

**Authors:** Laura Montoro-Dasi, Laura Lorenzo-Rebenaque, Ana Marco-Fuertes, Santiago Vega, Clara Marin

**Affiliations:** Departamento de Producción y Sanidad Animal, Salud Pública Veterinaria y Ciencia y Tecnología de los Alimentos, Facultad de Veterinaria, Instituto de Ciencias Biomédicas, Universidad Cardenal Herrera-CEU, CEU *Universities*, Calle Santiago Ramón y Cajal 20, 45115 Alfara del Patriarca, Valencia, Spain; laura.montoro@uchceu.es (L.M.-D.); laura.lorenzorebenaque@uchceu.es (L.L.-R.); ana.marcofuertes@uchceu.es (A.M.-F.); s.vega@uchceu.es (S.V.)

**Keywords:** *Salmonella* Infantis, poultry, One Health

## Abstract

*Salmonella* spp. has been globally recognized as one of the leading causes of acute human bacterial gastroenteritis resulting from the consumption of animal-derived products. *Salmonella* Enteritidis, *S.* Typhimurium, and its monophasic variant are the main serovars responsible for human disease. However, a serovar known as *S*. Infantis has emerged as the fourth most prevalent serovar associated with human disease. A total of 95% of isolated *S*. Infantis serovars originate from broilers and their derived products. This serovar is strongly associated with an elevated antimicrobial (AMR) and multidrug resistance, a resistance to disinfectants, an increased tolerance to environmental mercury, a heightened virulence, and an enhanced ability to form biofilms and attach to host cells. Furthermore, this serovar harbors genes that confer resistance to colistin, a last-resort antibiotic in human medicine, and it has the potential to acquire additional transferable AMR against other critically important antimicrobials, posing a new and significant challenge to global public health. This review provides an overview of the current status of the *S*. Infantis serovar in the poultry sector, focusing on its key virulence factors, including its virulence genes, antimicrobial resistance, and biofilm formation. Additionally, novel holistic strategies for controlling *S.* Infantis along the entire food chain are presented in this review.

## 1. Introduction

### 1.1. Historical Relevance of Salmonella spp. in Public Health: Past and Present

*Salmonella* spp. has been globally recognized as one of the leading causes of acute human bacterial gastroenteritis resulting from the consumption of animal-derived products, particularly those derived from the poultry and pig sectors [1].

This bacterial species is characterized as a gram-negative, rod-shaped, motile bacilli, belonging to the family *Enterobacteriaceae*, and exhibiting facultative anaerobic properties [2]. Additionally, these bacteria possess a diverse array of genetic mechanisms that contribute to their ability to colonize, adhere to, invade, and proliferate within, host cells. These mechanisms include the fimbriae, capsule, flagella, and toxins, and the virulence genes organized in their chromosome, such as pathogenicity islands, virulence plasmids, acid tolerance proteins, etc. [3,4].

In humans, the clinical symptoms of salmonellosis are primarily associated with self-limited gastroenteritis, characterized by diarrhea, abdominal pain, fever, headache, nausea, and/or vomiting, which typically resolve within 2 to 7 days. However, in certain cases, especially among children and elderly patients, the illness can progress to a severe and life-threatening condition, accompanied by systemic bacteremia [5]. In contrast, subclinical infections are common in animals, where the bacteria can easily spread between flocks without detection, and animals may become intermittent or persistent carriers [6].

Regarding the origins of human infection, there are multiple sources of *Salmonella* (*S.*), including foodborne contamination from a wide range of domestic and wild animals and vegetables, as well as direct contact with infected animals, contaminated environments, and humans [6,7,8,9,10]. However, the correlation between the *Salmonella* serovars found in humans and broilers is an aspect that is important to understanding the transmission and source of *Salmonella* infections in humans. Historically, there has been a recognized link between certain *Salmonella* serovars prevalent in broilers, and human infections. This is primarily due to the consumption of contaminated poultry products as a common route of transmission [11].

In the past, certain *Salmonella* serovars, such as *S.* Enteritidis and *S*. Typhimurium, were commonly associated with both human and broiler infections. These serotypes were frequently identified in poultry flocks, and contaminated meat and eggs were identified as significant sources of human infection. Consequently, since 2003, these bacterial species have been under scrutiny in Europe (Directive 2003/99/EC, modified in 2013) [12,13]. In 2004, the European Food Safety Authority (EFSA) published its first report, documenting a total of 192,703 confirmed cases of salmonellosis in humans. *S*. Enteritidis and *S*. Typhimurium were the most frequently identified serovars associated with human illness, with strong links to contaminated eggs and broiler meat [6]. These findings underscore the importance of implementing *Salmonella* National Control Programmes (SNCP) in each Member State (MS) (Directive 2003/2160/EC) [14].

Currently, this bacterium remains the second most prevalent zoonotic pathogen, following *Campylobacter*, responsible for gastrointestinal infections in humans. In 2021, there were 60,050 reported human cases. Furthermore, it ranks as the leading cause of foodborne outbreaks, with a total of 773 incidents (involving 6755 cases) reported during the same year. Among these cases, there were 11,790 hospitalizations, and 71 reported deaths [15]. In terms of the most significant serovars, the top five serovars responsible for human infections are currently *S*. Enteritidis, *S*. Typhimurium, *Salmonella* Typhimurium monophasic variant (mST), *S*. Infantis, and *S*. Derby. These serovars accounted for approximately 54.6%, 11.4%, 8.8%, 2.0%, and 0.93% of the reported cases in the European Union (EU) in 2021, respectively [15].

**Figure 1:**
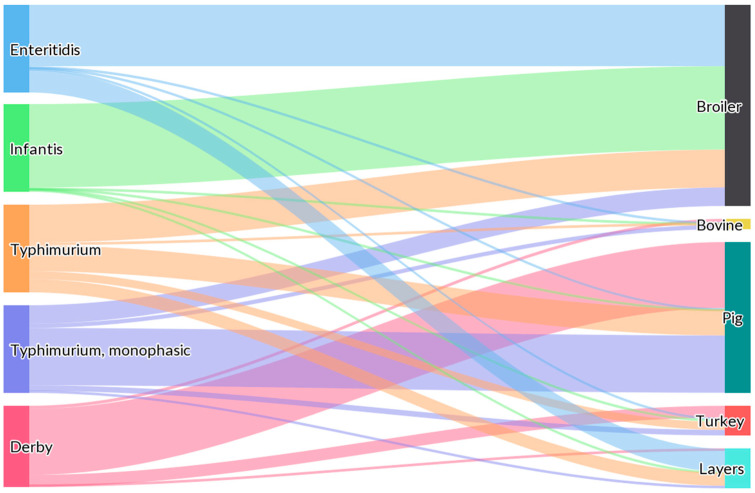
Sankey diagram showing the distribution of the most important European *Salmonella* serovars involved in human salmonellosis cases, reported from specified food-animal categories, by food-animal source in 2021. According to the EFSA report, animal and food data from the same source were merged: ‘broiler’ includes isolates from broiler flocks and broiler meat, ‘bovine’ includes isolates from bovines for meat production and from bovine meat, ‘pig’ includes isolates from fattening pigs and pig meat, ‘turkey’ includes isolates from fattening turkey flocks and turkey meat and ‘layers’ includes isolates from laying hen flocks and eggs. The width of the coloured bands linking the sources and serovars is proportional to the percentage of isolates of each serovar from each source. Obtained from: EFSA and ECDC, 2022 [15].

Moreover, if we examine the latest findings published by EFSA regarding the serovar distribution at the primary-sector level, it is evident that most *Salmonella* spp. serotyped isolates are derived from broiler production (*Gallus gallus domesticus*) (55.7%), followed by turkeys (*Meleagris gallopavo*) (12.9%), pigs (*Sus scrofa domestica*) (7.6%), and laying hens (*Gallus gallus domesticus*) (6.0%), as they were obtained from poultry populations covered by the SNCP [15]. As shown in Figure 1, *S*. Enteritidis was primarily related to broiler flocks and meat (70.0%), and to laying flocks and eggs (26.0%), whereas *S*. Infantis was strictly related to broiler sources (95.2%). Conversely, *S*. Typhimurium and mST isolates were mainly related to pig sources (43.2% and 65.4%, respectively) [15].

As reported above, *S*. Infantis is one of the serovars of *Salmonella enterica* that has been associated with both human and broiler infections. In fact, contaminated poultry products, including raw chicken meat and eggs, have been recognized as a common source of human infections with this serovar [16].

The correlation between *S*. Infantis in humans and broilers can be attributed to the transmission of the bacterium through the food chain [17]. The consumption of undercooked or improperly handled poultry products has been a significant risk factor for *S*. Infantis infections in humans. In recent years, there has been increased attention and effort given to also controlling *S.* Infantis in broiler production, and reducing its impact on human health. However, it is important to note that the prevalence of the different *Salmonella* relevant serovars, including *S*. Infantis, can vary geographically and over time. Continuous monitoring, surveillance, and research are necessary to assess the current situation, and identify any emerging trends or challenges associated with *S*. Infantis infections in both broilers and humans. Moreover, it is imperative to consistently maintain control measures, such as vaccination programs and stringent biosecurity strategies, in order to prevent the dissemination of pathogens and their antimicrobial resistance (AMR) throughout the food chain. These measures should not be diminished or relaxed [15].

### 1.2. Salmonella National Control Programmes in European Member States

SNCPs in the EU aim to implement comprehensive strategies for the prevention and control of, and reduction in, *Salmonella* contamination, including food production, animal health, and public health. These programs typically involve surveillance, monitoring, and management measures to identify and address potential sources of *Salmonella* contamination, enhance biosecurity practices, implement hygiene measures, and promote the responsible use of antimicrobials.

In 2007, the European Commission (EC) introduced a series of proposals for standards, with the goal of reducing the prevalence of *Salmonella* strains that pose a risk to both animals and humans. These standards were implemented to address the zoonotic nature of *Salmonella*, and mitigate its impact on public health [6]. Thus, the fundamental guidelines outlined a work program centered on assessing the current status of *Salmonella* in livestock farms within each sector. This assessment subsequently led to the establishment of specific community objectives. Following this, each country was required to develop a tailored control and eradication program for these pathogens, considering the unique characteristics of each member state (MS). Subsequently, depending on the production type (laying hens, broilers, or turkeys) and the intended commercialization of animals and products, documentation indicating the *Salmonella* status of the flock was required to accompany the sale. Ultimately, the trade of animals and derived products hinged on the absence of certain *Salmonella* serovars.

According to the EC proposals, the prevalence of *Salmonella* in livestock was studied in a sequential manner, starting with breeder farms, and subsequently extending to include laying hens, broilers, and turkeys. In 2008, the prevalence of *Salmonella* in pigs was also investigated. However, despite the detection of a high prevalence of *Salmonella* spp., pigs were ultimately excluded from the European *Salmonella* control plans.

The results obtained from the various prevalence studies unveiled high levels of *Salmonella* prevalence across Europe, highlighting significant variations among different MS (Table 1).

The official results were disheartening, prompting the establishment of rigorous community objectives for *Salmonella* control throughout Europe, via the SNCP. The primary serovars targeted under the SNCP for laying hen, broiler, and turkey production were *S.* Enteritidis and *S*. Typhimurium, which are the two serovars of utmost public health significance. The objective for laying hens was to achieve a 2% prevalence rate for *S*. Enteritidis and *S*. Typhimurium, rendering it impossible to sell fresh eggs for human consumption from positive flocks since 2009. In 2011, the monophasic *S*. Typhimurium serovar was included in the SNCP. For broiler and turkey flocks, a longer-term and more ambitious goal of attaining a 1% prevalence rate for *S*. Enteritidis and *S*. Typhimurium was set. Lastly, for breeder flocks, the objectives were even more stringent, encompassing not only *S*. Enteritidis, *S*. Typhimurium, and their monophasic variant, but also *S*. Hadar, *S*. Virchow, and *S*. Infantis, within the SNCP. In the event of any of these serovars being detected at the farm level during official or self-imposed controls mandated by the EU, all birds within the flock would be culled, and their by-products would be destroyed [6].

In this context, the European poultry sector began to intensively control the challenge posed by *Salmonella* spp., implementing crucial biosecurity measures, and adopting good management practices. As a result, most European MSs have aligned themselves with the community objectives set forth by the EC.

**Figure 2:**
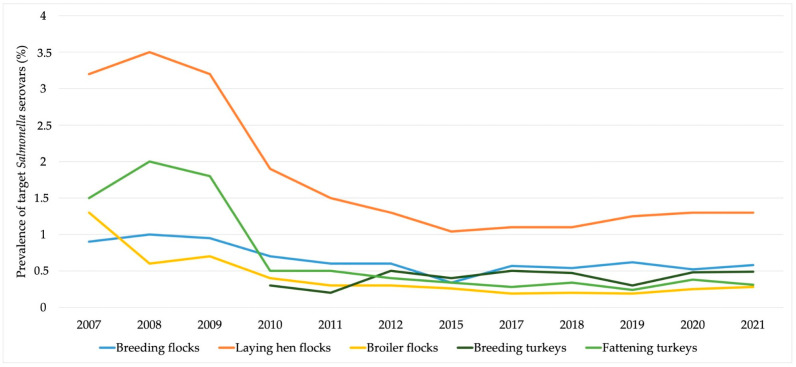
The evolution of the prevalence of target *Salmonella* serovars in the European poultry production sector. Data obtained from the European Food Safety Authority reports [6,15,23,24,25,26,27,28,29,30,31,32].

As a result of all efforts performed in the poultry production sector, the prevalence of *Salmonella* target serovars in the European Union has been gradually decreasing (Figure 2). Moreover, the latest data reported by EFSA on the prevalence of *Salmonella* in poultry and pigs are presented in Table 2.

Data obtained from the European Food Safety Authority (EFSA) and European Centre for Diseases Prevention and Control (ECDC), 2022 [15].

### 1.3. Salmonella Infantis: The Current Challenge

Until 2010, various dominant serovars were prevalent in broiler production, varying by country, company, and other factors. Moreover, as reported above, the prevalence of the target serovars in the poultry sector (*S.* Enteritidis and *S.* Typhimurium) has remained under the European Union objective’s limit (1%) since 2008 [23]. However, from 2010 onwards, there has been a significant surge in the prevalence of *S*. Infantis. Since 2014, *S*. Infantis has become the primary serovar isolated in broiler production in many MSs. Presently, broilers and their derived products account for 95% of *S*. Infantis isolates [15].

Different hypotheses could explain the surge in this serovar in broilers. One possibility is the implementation of vaccination and control programs targeting *S*. Enteritidis and *S*. Typhimurium, which have led to a decrease in, or even elimination of, these serovars on farms [3]. This reduction created an ecological niche, which allowed the proliferation and spread of *S*. Infantis, particularly in broiler production, where it accounts for half of all *Salmonella* isolates. Notably, *S*. Infantis has been found to possess various genetic strategies that enhance its epidemiological fitness. These strategies include the acquisition and transmission of AMR, a resistance to heavy metals, the presence of mobile virulence genes, and the ability to form biofilms [3,4,22,23,24,25,26,27,28,29,30,31,32,33,34,35].

Different virulence factors have been identified in *S*. Infantis, but one of the most significant is the *pESI*-like mega-plasmid (the plasmid of emerging *Salmonella* Infantis). This plasmid was initially discovered in Israel in 2014 [36], and has since spread globally, particularly in the European Union and the United States [35].

These genes are linked to an increased AMR and multidrug resistance (MDR), resistance to disinfectants, a higher tolerance to environmental mercury, enhanced virulence, and an improved ability to form biofilms and attach to host cells [33,35]. Thus, this is one of the reasons why *S*. Infantis is known for its high persistence in broilers. Furthermore, the presence of *extended-spectrum-β-lactamase* (ESBL) genes, and the *mcr-1* (mobilized colistin resistance) gene, has been reported in this plasmid, providing the bacteria with resistance to colistin, which is considered a last-resort antibiotic in human medicine [37]. Additionally, it enables the acquisition of additional transferable AMR against other critically important antimicrobials [33,37].

Outer membrane porin proteins also play a crucial role in regulating membrane permeability, and facilitating the diffusion of hydrophilic antibiotics, including β-lactams, into bacterial cells. However, a reduction in membrane permeability is a common mechanism of antibiotic resistance, as it effectively hinders the entry of antibiotics. *S.* Infantis can employ various strategies to achieve this, such as selectively decreasing the abundance of pore proteins in the outer membrane, altering the size of porin proteins such as *OmpF* and *OmpC*, and increasing the thickness of the cell wall. These adaptations collectively contribute to a reduced membrane permeability, thereby promoting a high level of antibiotic resistance [38,39]. Moreover, efflux pumps, located in the bacterial cell membrane, act as transport proteins that can actively remove toxic substances from within the cell, either in a selective or non-selective manner. When efflux pumps are overexpressed, they hinder the accumulation of antibiotics inside bacterial cells, leading to significant levels of antibiotic resistance. Efflux pumps associated with antibiotic resistance have been extensively studied in foodborne pathogens (including *S*. Infantis), with approximately 24% of the literature published in the last five years focusing on this topic. This highlights the significant attention given to efflux pumps as one of the most extensively researched mechanisms of antibiotic resistance [38].

Furthermore, Lapierre et al. (2020) reported a 100% prevalence of genes associated with invasion (*invA*, *sipA*, *sipD*, and *sopD*), intracellular survival (*SEN1417*, *mgtC*), and biofilm formation (*pagK*) in *S*. Infantis strains isolated from chicken meat in Chile. Additionally, they identified various resistance determinants linked to mobile genetic elements, such as the *blaCTX-M 65* and *qnrB* genes. Furthermore, *S*. Infantis prophages (bacteriophages hidden within the bacterial genome, which can be active, or remnants of active prophages) can harbor additional AMR and MDR genes, along with virulence genes. This genetic combination confers a competitive advantage to the bacteria in their interaction with the host [40,41,42].

**Figure 3:**
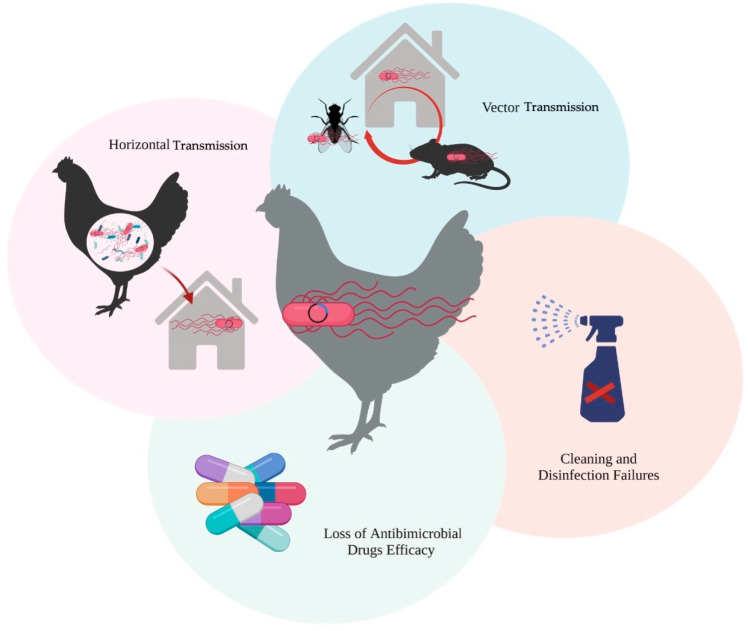
Predisposal factors in the maintenance and transmission of *S*. Infantis *pESI*.

On the other hand, it is important to emphasize that AMR represents one of the most significant threats to global public health, and is a major concern for consumers [43]. This resistance emerges as bacteria and other microorganisms evolve over time, acquiring the ability to overcome the effectiveness of medicines intended to eliminate them. Consequently, treating infections becomes more challenging, and there is an increased risk of disease transmission, severe illness, and mortality. Consequently, antimicrobial drugs lose their effectiveness, leading to persistent infections [44,45,46]. Additionally, studies using a mouse model have demonstrated that *pESI* can be transferred from *S*. Infantis to gut bacteria or other *Salmonella* serovars, through horizontal gene transfer during co-infection [47]. This implies that MDR and enhanced virulence can potentially be disseminated to other pathogens, as well (Figure 3).

Over the past few years, the rising occurrence of *S.* Infantis infections in both humans and animals has been further complicated by the dissemination of multidrug-resistant clones in various countries. These MDR strains have been associated with prolonged illness, extended hospital stays, and higher mortality rates, posing significant public health concerns [34]. Furthermore, there has been an increase in AMR among *S*. Infantis strains circulating in the poultry industry. It has been well-documented that this serovar possesses a remarkable ability to acquire and disseminate AMR and MDR genes within the environmental microbiota. In fact, the latest AMR monitoring report by EFSA revealed that *S*. Infantis strains exhibited significantly higher rates of resistance to sulfonamides, tetracyclines, and critical antibiotics, including ciprofloxacin, cefotaxime, and their combinations, compared to other *Salmonella* serovars. A predominant clone in European broiler production was found to be resistant to ciprofloxacin/nalidixic acid, sulfamethoxazole, and tetracycline. Additionally, a considerable percentage of *S*. Infantis strains (45.3%) demonstrated MDR, and the production of ESBL enzymes was also frequently observed [4,15,35,42,48,49].

Additionally, studies on *Salmonella* biofilm formation have reported varying degrees of biofilm-forming ability in *S*. Infantis, ranging from moderate to very high. This ability is associated with the persistence of infections on farms, indicating that *S*. Infantis has the capacity to form robust biofilms that contribute to its ability to survive and persist in farm settings [50,51,52,53]. Biofilms are defined as communities of surface-attached bacteria, and are considered a defensive strategy that enhances microbial tolerance to chemical, physical, and biological agents [52,54]. The formation of a biofilm is developed in five phases: phase 1, the reversible adhesion of the bacteria to the surface; phases 2 and 3, irreversible adhesion to the surface, and division, with the production of a protective exopolymer, and the final colony development, with the dispersal of colonizing cells; phase 4, the growth and maturation of the adherent cells; and phase 5, the diffusion of the colonizing cells. Eventually, some bacteria in the biofilm matrix are released from the biofilm, to colonize new surfaces. It is important to highlight that bacteria living in biofilms can be up to 1000 times more resistant to commonly used disinfectants in livestock, compared to their planktonic form [50,55].

Moreover, within the biofilm, bacteria can employ various survival strategies to evade the host’s defense systems. They can adapt to poor environments by altering their metabolism, gene expression, and protein production. Through these adaptive mechanisms, bacteria can also develop an increased resistance to antimicrobial therapy, by inactivating antimicrobial targets, or reducing the cellular functions that antimicrobials disrupt [53,56].

Finally, when a strain of *S*. Enteritidis, *S*. Typhimurium, or mST is isolated from laying hen or broiler farms, the birds must be culled, and their enclosures must undergo thorough disinfection before a new flock is introduced, as per European regulations. However, since *S*. Infantis is not included in the SNCPs, if an *S*. Infantis strain is isolated at the farm level, these animals continue throughout the production chain, and the competent authorities do not oversee the cleaning and disinfection procedures. This situation allows the bacteria to persist in the farm’s environment, and facilitates their transmission to future flocks.

## 2. Methodology

An systematic literature search was conducted, in which different sources of information were reviewed and evaluated, to address the past, present, and future of *Salmonella* epidemiology in the poultry sector, specifically for the serovar *S*. Infantis, and to expose the most innovative, effective, and environmentally and animal-friendly control tools at the field level.

The search was performed using search engines such as PubMed, Google Scholar, and Scopus. In addition, reports and publications by health organizations such as the World Health Organization (WHO), the European Food Safety Authority (EFSA), the European Centre for Disease Prevention and Control (ECDC), the European Medicines Agency (EMA), and European legislation (EUR-Lex) were reviewed.

This review included scientific information and legislation available in English, and published since 2003. The search terms that provided most of the information were: “Salmonella”, “Salmonella Infantis”, “Antimicrobial resistance”, “poultry”, “broiler”, “biosecurity”, “cleaning and disinfection”, “bacteriophages”, “microbiota modulation”, “additives”, “probiotics”, “prebiotics”, and “vaccination”.

The titles and abstracts of the identified records were screened, and the references from the full-text searches deemed relevant were also screened, and included if relevant. Finally, all the information recovered was structured, and the manuscript was written.

## 3. Innovative Control Tools in Development against *S.* Infantis

Historically, the most effective methods for controlling *Salmonella* spp. at the farm level have been biosafety measures and good practice. Key factors for success include thorough cleaning and disinfection, with special attention given to using effective detergents, the complete removal of organic matter, the appropriate concentration of disinfectants, and the quality of the cleaning water. Additionally, pest control, through bait rotation and continuous monitoring; the strict and continuous sanitation of drinking water throughout the production cycle; vaccination using a combination of live and inactivated vaccines; and the use of various food tools, such as symbiotics and acids have all played crucial roles.

Despite the these tools’ success in maintaining bacterial control in accordance with European standards, they have not been able to prevent the spread of the *S*. Infantis serovar in broilers and its derived products, as reported by the ECDC and EFSA in 2022. Therefore, there is a need to develop and implement, at the field level, novel, cost-effective, and efficient tools, such as the inclusion of bacteriophages in cleaning and disinfection protocols; the study of microbiota in situ, to make effective decisions about its modulation; and the use of new vaccine development technologies to obtain protection against the colonization of *S*. Infantis from the beginning of the poultry sector chain. This way, it could be possible to protect chickens and their environment from a holistic point of view, to reduce the prevalence of this serovar throughout the food chain.

### 3.1. Cleaning and Disinfection Combined with Bacteriophage Products against Persistent S. Infantis Strains

*Salmonella* can be transmitted in the poultry sector through vertical, pseudo-vertical, or horizontal transmission. Additionally, due to intermittent excretion, *Salmonella*-positive flocks can spread the bacteria to various farm facilities, as reported by the EFSA and ECDC, 2019 [19]. Subsequently, *Salmonella* can persist and potentially multiply in residual organic matter, as observed in studies by Gosling et al., 2016 [57]. This perpetuates the infection, and facilitates transmission between different flocks. Therefore, it is essential to establish precise cleaning and disinfection protocols on poultry farms, to mitigate the risk of *Salmonella* contamination [58].

Moreover, in contrast to other important *Salmonella* serovars (mainly *S.* Enteritidis and *S.* Typhimurium), which can usually be eliminated within a single or a few flock cycles, farms persistently infected with *S.* Infantis are constantly reported [59], indicating that this serovar might be more resistant to commonly used disinfectants, based on an increased biofilm-forming ability, and a higher tolerance to thermal, acid, and osmotic stress, as reported above [59,60]. A combination of interventions is necessary to prevent the further spread and persistence of *Salmonella* on livestock premises. Among these interventions, the effective terminal cleaning and disinfection of poultry facilities following the depopulation of an infected flock play a crucial role in eliminating *Salmonella* from poultry farms [61].

In this context, among the traditionally available disinfectants for livestock surface hygiene, such as quaternary ammonium compound products, iodine-based compounds, chlorocresols, and peracetic-acid-based compounds, formaldehyde has been proven to be one of the most effective disinfectants against zoonotic pathogens, including *Salmonella* [62]. However, due to its classification as carcinogenic, mutagenic, and highly toxic, according to EU Regulation 605/2014 [63], the use of formaldehyde is restricted in the EU. This has created an urgent need to explore alternative methods for farm hygiene [62]. Moreover, it has also been demonstrated that standard cleaning and disinfection protocols, which involve the use of orchard sprayers in applying disinfectant within poultry facilities, are not effectively eliminating contamination from within the affected poultry facilities [61]. 

The survival of *S*. Infantis after cleaning and disinfection procedures is particularly significant in the drinker and feeder lines, the anteroom (including electrical panels, floors, and surfaces), farmers’ boots, and fomites, as well as the farm’s surrounding environment [61]. For this reason, the development of new cleaning and disinfection products that are both effective, and environmentally friendly, is crucial. In recent years, research has focused on the efficacy of bacteriophage application as a sanitizer, particularly in the food industry and farming, with promising results [47,59]. Bacteriophages are viruses that specifically infect bacterial cells, as their life cycle is intricately linked to prokaryotic cells [64,65]. These viruses are highly specific, self-replicating, self-limiting, well-tolerated, and accessible from multiple sources [66,67]. In fact, they are ubiquitously distributed, based on the presence of their host bacteria, and are found in water, soil, air, plants, humans, and other animals, and are therefore consumed by people [65,68]. Bacteriophages are viruses that specifically infect bacterial cells, as their life cycle is intricately linked to prokaryotic cells [64,65,69].

Bacteriophage-based products can be employed as biosanitizers in hatcheries, farms, transport crates, and poultry processing plants, and on food-contact surfaces [64]. For instance, a study conducted in Spain demonstrated the high efficacy of phage application in poultry farm facilities, achieving a 100% efficacy rate against persistent *Salmonella* strains [60]. In fact, it has been demonstrated that the aerosol spraying of poultry and litter in production facilities can effectively prevent the horizontal transmission of the pathogen. Moreover, bacteriophages have shown effectiveness in inhibiting biofilm formation, and dispersing mature biofilms produced by pathogenic bacteria on surfaces commonly found in the poultry industry [64]. It is important to highlight that the use of bacteriophages in Europe is currently not authorized. However, the EC is investing significant funds in research groups to study the viability of bacteriophage application in the agri-food industry, from farm to fork. In other countries, such as the United States or Russia, bacteriophages are widely used at the farm level, as food additives, or in human therapy [70,71].

Finally, bacteriophage therapy is also considered safe and especially useful in the control of zoonotic bacteria [72,73,74,75]. However, there are still no bacteriophage-based tools specifically developed against *S.* Infantis that are authorized in the EU.

### 3.2. Microbiota Modulation to Control S. Infantis during the Production Cycle

Microbial communities are commonly defined as the assemblage of microorganisms living together (including commensal, symbiotic, and pathogenic assemblages), with their interactions in a common biome. Furthermore, it has been demonstrated that the composition and development of the microbiota have a significant influence on animal health, productivity, and disease control [76], mainly due to the effect on its physiology, nutrient exchange, exclusion of pathogens, and also its modulation of the immune system [77,78]. Indeed, the microbiota play a crucial role in modulating both the innate and acquired immune responses of broilers. In terms of the innate immune response, the intestinal mucosa serves as the primary defense against infections. Within the acquired immune system, commensal bacteria contribute to the protection of the mucosal membrane, by regulating the immune response. They control the secretion of mediators by cells of the acquired immune system, and stimulate helper T cells, thus promoting a balanced and effective immune response [77,79,80]. In this line, the microbial communities present in the normal intestinal microbiota play a significant role in preventing the colonization of bacterial pathogens in chickens, a phenomenon known as “colonization resistance”. This resistance includes both competitive exclusion, and immune modulation in young chicks [81]. Thus, manipulating the intestinal microbiota has emerged as an intriguing strategy for preventing intestinal infections, and promoting the overall health and performance of chickens in the production setting.

Traditionally, in animal production, the control of pathogens, and modulation of the intestinal microbiota were achieved through the use of antibiotics at sub-therapeutic doses, commonly known as growth promoters. However, due to the emergence of, and increasing concern around, AMR in public health, the use of antibiotics as growth promoters was banned in the EU in 2006 [82]. Therefore, there is a need to develop innovative, effective, and sustainable solutions that allow for the modulation of the intestinal microbiota. This is essential to promote the development of resilient animals, and to prevent the presence of pathogens or zoonotic microorganisms, including *Salmonella* [83].

Diverse strategies and approaches exist when it comes to modulating the intestinal microbiota with the aim of enhancing their competitiveness [81,84]. In practice, various methods are commonly employed, such as the administration of competitive exclusion (CE) cultures, probiotics, prebiotics, symbiotics, and organic acids, as well as bacteriophages and fecal transplants, that could decrease bacterial pathogens, or promote the growth of beneficial bacteria. While each of these strategies has specific applications and limitations within the context of food-animal production, they provide a natural means to regulate the intestinal microbiota, ensuring a healthy microbial community dominated by beneficial bacteria, and ultimately enhancing the host’s performance and wellbeing [81].

Nurmi and Rantala (1973) developed the initial investigation that served as a fundamental reference for numerous studies exploring the use of probiotics, and manipulation of the microbiome, in chickens [85]. In their study, samples obtained from the crop and intestinal tract of healthy roosters were directly introduced into the crop of newly hatched chicks. Subsequently, the chicks were infected with *S.* Infantis at two different inoculum levels. This treatment consistently and effectively decreased the presence of *S*. Infantis in the crop, small intestine, and ceca of the experimentally infected birds [85].

A wide range of feed additives aim to promote the growth of beneficial bacteria, such as *Lactobacillus* spp. and *Bifidobacterium* spp., while reducing the presence and colonization of pathogenic or zoonotic bacteria, such as *Salmonella*. Numerous studies have demonstrated their effectiveness in achieving these objectives. Additionally, these additives have demonstrated the ability to modulate the immune response, and enhance the efficacy of *Salmonella* vaccination [67,86,87,88,89].

However, studies focusing on tools developed to reduce *S*. Infantis infections are scarce, but some studies have shown the efficacy of *Lactobacillus fermentum* in chickens affected by this *Salmonella* serovar. These animals showed an improvement in the height of the intestinal villus (destroyed by *Salmonella*) throughout the gut and, thus, an improvement in the surface area for nutrient absorption. In addition, there was an improvement in serum immunoglobulin levels [90]. Moreover, the introduction of probiotic strains has been shown to decrease the levels of *S.* Infantis in the gastrointestinal tract of broiler chickens. In fact, El Hage et al. (2022) observed that *Ligilactobacillus salivarius* 16/c6 was able to significantly exclude the adhesion of *S*. Enteritidis, *S*. Infantis, and *S*. Kentucky to the cell culture [91]; and Schneitz et al. (2016) determined that Broilact (a CE product) was the sole treatment substance, compared with FloraMax-B11 and Colostrum Liquido, capable of establishing itself within the gut of newly hatched chickens in a manner that effectively prevented the colonization of *S.* Infantis [92].

Other natural additives have been tested for their ability to prevent *Salmonella* colonization, including essential oils. Di Vito et al. (2020) showed the efficacy of *Origanum vulgare* essential oil, with a mixture of feed additives used in feed as flavorings, in reducing microbial adhesion to intestinal target cells, and in reactivating the sensitivity of multi-resistant *Salmonella* spp. strains to ciprofloxacin, one of the most used antibiotics in veterinary practices [93].

Regarding the use of nutritional strategies, Jha et al. (2019) observed that the inclusion of wheat bran with a reduced particle size, and also derived components such as arabinoxylooligosaccharides or other fiber types, including fructooligosaccharides and mannan oligossacharides, reduced *Salmonella* colonization in vivo and in vitro [94], according to other studies [94,95,96,97,98,99,100,101]. Moreover, a reduction in *Salmonella* colonization on a wheat-based basal diet supplemented with xylanase has been reported [102]. In this way, fermentable fibers have garnered research interest as potential sources of microbiome modulators. However, it appears that certain fractions of cereal grains can be a source of several β-glucans, which are also potentially fermentable, resulting in the antagonism of pathogens such as *Salmonella* [103]. Furthermore, recent studies have observed that other dietary fibers, such as long-chain glucomannan, can reduce the colonization of *Salmonella* in the intestines of birds, thereby reducing the excretion of the bacteria into the environment, while also stimulating the immune response of the animal [104].

Other tools include organic acids. In fact, short-chain carboxylic acids are considered the most commercially useful for feed additives, including formic, propionic, and butyric acids. They are employed to decrease the luminal pH, and improve gut health, by providing carbon sources for villi growth, promoting the growth of beneficial bacteria (*Lactobacillus* and *Bifidobacterium*), and decreasing harmful bacteria, such as *Salmonella* [103,105]. Moreover, alternative compounds, such as anthocyanins, have not only demonstrated a reduction in *Salmonella* colonization in the liver and spleen of affected chickens, but also decreased the secretion of inflammatory cytokines, up-regulated the expression of ileal genes encoding mucosal and tight junction proteins, and modulated the composition of the cecal microbiota [106]. However, it is important to note that serovar and strain differences are crucial factors in the survival capability of *Salmonella* in feeds, influencing their respective responses to the presence of acids added [103,105]. Nevertheless, exposure to these additives has also been reported to lead to changes in the virulence expression in the bacteria, which could in turn impact the pathogenesis expression levels [103,107,108,109].

Finally, fecal microbiota transplantation is a unique approach that involves the transfer of a complete bacterial population, preserving the intricate interactions between microorganisms, including nutrient competition and metabolite exchange, which are crucial for successful bacterial colonization. In the case of poultry, studies have shown that transplanting digestive material from adult animals to 1-day-old chicks enhances bacterial diversity, provides greater protection against *Salmonella* spp. colonization, and promotes the healthy development of the digestive and locomotor systems. The changes in microbial composition and the resulting metabolites exert their effects throughout the production cycle. Although fecal transplantation is a novel alternative, it is considered a promising tool in the poultry industry, and is currently being studied and developed to optimize its use and achieve favorable outcomes [110].

Traditionally, in order to address these changes, culture-dependent methods have been employed for microbiota studies. However, in recent years, there has been a rapid development in culture-independent technologies. Over their evolution, these technologies have not only improved in their taxonomic resolution capabilities, but have also become more affordable and user-friendly. Among these advancements, the MinION has emerged as a prominent tool in the field [111,112,113]. The MinION, developed by Oxford Nanopore Technologies, is a compact and portable sequencer. With the MinION, there is an attempt to address one of the main limitations of other culture-independent methods, such as 16S gene sequencing, which is the ability to accurately identify species on-site [113,114]. In recent years, Kürekci et al. (2021) employed MinION for the genomic characterization of *S.* Infantis isolated from raw chicken meat samples sold in Turkey. This study showed the widespread dissemination of the pESI-like megaplasmid in *S.* Infantis strains of chicken meat [115]. Similarly, Egorova et al. (2022) also used MinION to determine the characteristics and traits of MDR *S.* Infantis isolates of food origin, from Russia. The authors detected genes of aminoglycoside-modifying enzymes, and genes responsible for resistance to tetracycline, sulfonamide, and chloramphenicol in the isolated *S.* Infantis. The development of these technologies and their application in the field can potentially represent a significant advancement in the early detection and rapid understanding of the status of *S*. Infantis and its genes, in animals and products [116].

### 3.3. Vaccination as Prevention Tool against S. Infantis in Poultry

Vaccination is a safe and effective way of protecting animals against diseases. This tool utilizes natural defenses to build resistance to specific infections, and to strengthen the immune system [117]. Vaccines prevent disease by training the immune system to recognize and respond to a pathogen efficiently, thereby reducing the severity of the disease, or even preventing infection altogether. Additionally, vaccines play a crucial role in reducing the need for antibiotic therapy. As a result, this control tool not only protects against pathogens, but also helps to curb the spread and development of AMR [118,119]. Against this background, vaccination emerges as one of the most crucial control tools for *Salmonella* in poultry production. As a result, several studies have been conducted to develop safe and effective vaccines in this sector.

Its protection effect depends on the immune response of the host to different antigenic components. These antigens stimulate humoral and cell-mediated immunity. On the one hand, B cells (together with neutrophils, macrophages, and neutrophils) use surface immunoglobulins as antigen receptors, and differentiate into plasma cells, able to secrete antibodies (IgM, IgG/IgY, and IgA). On the other hand, T cells differentiate to CD4^+^ cells (T-helper cells) and CD8^+^ cells (T-cytotoxic cells), creating immunological immunity to fight efficiently against future infections [120].

There are different techniques for developing bacterial vaccines, resulting in various types of vaccine. It is important to note that bacteria, unlike viruses, are complex microorganisms with intricate genomes that encode hundreds of proteins, and possess mechanisms that facilitate evasion from the host’s immune cells [121]. For this reason, different vaccines presentations have been developed. On the one hand, there are commercially available live attenuated vaccines, or inactivated (killed) vaccines, both including the whole bacterium [120]. On the other hand, it is also possible to inoculate metabolic bacterial products (toxoids), purified parts of bacteria (subunit vaccines), or recombinant vector vaccines, to generate immunity in animals [122,123,124,125].

In this context, different research groups are currently developing and evaluating an effective vaccine against *S*. Infantis in poultry production, as well as testing cross-protection within different serovars. On the one hand, there are different studies being conducted to develop and test attenuated or inactivated vaccines against *S*. Infantis. Varmuzova et al. (2016) created a live attenuated trivalent vaccine against *S*. Enteritidis, *S*. Typhimurium, and *S*. Infantis, administered orally to day-old chicks. However, the vaccine’s efficacy in protecting against *S*. Infantis colonization in the liver and ceca was not effective [126]. Moreover, MSD Animal Health developed an inactivated trivalent bacterin, adjuvanted with aluminum hydroxide gel, targeting *S*. Enteritidis, *S*. Typhimurium, and *S*. Infantis (Nobilis^®^ Salenvac ETC). The efficacy of this vaccine was tested in SPF day-old layers through double intramuscular administrations at 6 and 10 weeks of age. The results demonstrated a significant reduction in both intestinal colonization, and internal organ invasion, for the homologous serovars [126]. Subsequently, the same vaccine was evaluated in Ross 308 broiler breeders, where it was incorporated into the commercial vaccination program at 10 and 17 weeks of age. The study observed a significant decrease in both the fecal shedding of *Salmonella*, and the invasion of internal organs [127]. Likewise, Huberman et al. (2022) developed an inactivated trivalent vaccine with an oil adjuvant, which was administered to day-old layers at 8 and 11 weeks of age. The study demonstrated effective protection against *S*. Infantis colonization [128].

On the other hand, recombinant vaccines against *S*. Infantis are also being developed. In 2019, Müştak and Yardımcı (2019) reported that their aroA gene-deleted mutant of *S.* Infantis exhibited decreased adhesion and invasion abilities compared to the wild-type strain during in vitro tests. This finding suggests that the mutant strain could serve as a potential vaccine candidate [129]. However, no further results have been published regarding this vaccine. Additionally, another innovative approach to recombinant subunit vaccines is being studied, known as cochleate system vaccines. Cochleates are spiral structures formed by the interaction of anionic lipid vesicles with divalent cations, specifically phospholipid-calcium precipitates. They are used to protect and deliver bacterial compounds, such as membranes, proteins, or DNA, administered via oral routes, such as vaccines. In this regard, Avedaño et al. (2021) achieved encapsulated *S*. Infantis cochleates, and proposed such a novel prototype oral vaccine. Moreover, safety trials were performed, and no clinical or pathological lesions were observed after one dose administered at 6 weeks of age [130]. Subsequently, these researchers took a step further by creating a trivalent vaccine (targeting *S*. Enteritidis, *S*. Typhimurium, and *S*. Infantis) using similar technology. The efficacy of the vaccine was evaluated after a double-dose vaccination program at 6 and 9 weeks of age. The results demonstrated a significant decrease in cecal colonization after the challenge, indicating the vaccine’s effectiveness [131]. Finally, Maiti et al. (2022) developed a trivalent outer membrane vesicle (OMV) vaccine (*S*. Enteritidis, *S*. Typhimurium, and *S*. Gallinarum), with the aim of achieving long-term broad-spectrum protection against most prevalent *Salmonella* serovars, including *S*. Infantis. OMVs are considered as subunit acellular vaccines produced from the outer membrane of bacteria, possessing a similar structure to the bacterial membrane. Due to their resemblance, OMVs are regarded as potential immunomodulators against various pathogens, including *Salmonella*. In fact, by administering the OMV vaccine at 0, 14, and 28 days of age, a cross immunization against *S*. Infantis was successfully achieved after three doses [132].

Following the concept of cross-protection among *Salmonella* serovars, various commercially available vaccines designed against *S*. Enteritidis and *S*. Typhimurium have been tested for their effectiveness against *S*. Infantis. However, the results have not been promising thus far. Vaccines such as live *S*. Enteritidis/Typhimurium Avipro^®^ *Salmonella* Duo (ElancoTM) and Salmovac 440 SE (Ceva Santé Animale) have shown some reduction in *S*. Infantis colonization, but the level of protection achieved has been limited [133,134]. However, the modified live Poulvac ST vaccine (Zoetis Inc., Parsippany-Troy Hills, NJ, USA) has shown some limited efficacy in protecting against internal organ colonization by *S.* Infantis [135].

Table 3 provides an overview of the commercially approved and available vaccines against *Salmonella* in poultry, along with their main characteristics. There are four attenuated oral vaccines for *S*. Enteritidis, and two for *S*. Typhimurium. Additionally, there are three inactivated intramuscular vaccines, one attenuated oral vaccine for both *S*. Enteritidis and *S*. Typhimurium, and one available inactivated intramuscular vaccine for *S*. Enteritidis, *S*. Typhimurium, and *S*. Infantis. These vaccines need to be administered during the rearing phase, prior to the start of the production cycle, in breeders or layers. It is worth noting that, as shown in Table 3, there are currently no vaccines available specifically for broiler production (source: https://www.ema.europa.eu/en, accessed on 12 May 2023).

## 4. Conclusions and Future Perspectives on *Salmonella* Vaccination in Poultry

Poultry is a crucial sector in global food production, encompassing both layers and broilers, as it provides consumers from diverse countries and cultures with high-quality, nutritious, affordable protein. However, the industry continually faces new challenges that necessitate a “One Health” approach. Among these challenges, the presence of *Salmonella*, particularly the *S*. Infantis serovar, is of significant concern. This serovar exhibits persistence throughout the food chain, high levels of AMR and MDR, resistance to disinfectants, an increased tolerance of environmental mercury, enhanced virulence, and improved abilities to form biofilms and attach to host cells.

The evolution of bacteria toward more resistant strains, coupled with the growing consumer demand for animal welfare and sustainable production (including backyard systems), underscores the need to enhance *Salmonella* control measures. Currently, there is only one authorized vaccine against *S*. Infantis. Consequently, numerous research groups are actively working on developing candidate vaccines targeting this serovar, and investigating the efficacy of cross-protection with authorized vaccines for other serovars against *S*. Infantis.

In this context, integrating vaccination with rigorous cleaning, disinfection (including the application of bacteriophages for persistent strains), and pest-control protocols (to prevent recontamination of the premises), along with microbiota modulation (which primes the animals’ immune system for bacterial challenges), presents a novel holistic strategy to combat the persistent presence of *S*. Infantis in the meat-poultry sector. This approach recognizes the complex interplay between animal health, human health, and environmental health, aiming to ensure safe and sustainable poultry production.

## Figures and Tables

**Table 1:** First reported EU prevalence results of *Salmonella* spp. positive cases in poultry production (breeders, laying hens, broilers, breeding turkeys, and fattening turkeys), and in pig production (breeding pigs and fattening pigs).

Animal Production Type	EU Mean Prevalence (%)	Prevalence Rate in EU Member States (%)	References
**Breeding flocks**	2.9	0–26.3	[18]
**Laying hen flocks**	30.7	0–79.0	[19]
**Broiler flocks**	23.7	0–68.2	[20]
**Breeding turkeys**	13.6	0–82.9	[21]
**Fattening turkeys**	30.7	0–78.5	[21]
**Breeding pigs**	28.7	0–64.0	[22]
**Fattening pigs**	33.3	0–55.7	[22]

**Table 2:** The last reported European Union prevalence results of *Salmonella* spp. positive cases in poultry production (breeders, laying hens, broilers, breeding turkeys, and fattening turkeys), and in pig production (breeding pigs and fattening pigs).

Animal Production Type	EU Mean Prevalence (%)	EU Mean Prevalence for Target Serovars (%)	Prevalence Rate in EU Member States (%)
**Breeding flocks**	2.5	0.58	0–13.4
**Laying hen flocks**	3.3	1.3	0–22.9
**Broiler flocks**	3.8	0.28	0–21.7
**Breeding turkeys**	3.9	0.49	0–50.0
**Fattening turkeys**	9.1	0.31	0–42.9
**Pigs**	2.9	-	-

**Table 3:** Commercially available vaccines for *Salmonella* in European Union poultry production, according to the European Medicines Agency (https://www.ema.europa.eu/en, accessed on 12 May 2023).

Name	Company	Presentation	*Salmonella* Serovar	Production Type	Administration Route
AviPro Salmonella Vac E	Elanco GmbH	Attenuated	SE	Breeders and layers	Oral
Cevac Salmovac	Ceva Salud Animal	Attenuated	SE	Breeders and layers	Oral
Primun Salmonella E	CALIER, S.A.	Attenuated	SE	Breeders and layers	Oral
Nobilis SE	MSD Animal Health, S.L.	Attenuated	SE	Breeders and layers	Oral
AviPro Salmonella Vac T	Elanco GmbH	Attenuated	ST	Breeders and layers	Oral
Primun Salmonella T	CALIER, S.A.	Attenuated	ST	Breeders and layers	Oral
Nobilis Salenvac T	MSD Animal Health, S.L.	Inactivated	SE + ST	Breeders	Injection
Avian Secure	HIPRA, S.A.	Inactivated	SE + ST	Breeders and layers	Injection
Gallimune Se+St	Boehringer Ingelheim	Inactivated	SE + ST	Layers	Injection
AviPro Salmonella DUO	Elanco GmbH	Attenuated	SE + ST	Breeders and layers	Oral
Nobilis Salenvac ETC	MSD Animal Health, S.L.	Inactivated	SE + ST + SI	Breeders and layers	Injection

SE, *Salmonella* Enteritidis; ST, *Salmonella* Typhimurium; SI, *Salmonella* Infantis.

## Data Availability

Data sharing is not applicable to this article.
